# Characterization of Human Chromosomal Material Exchange with Regard to the Chromosome Translocations Using Next-Generation Sequencing Data

**DOI:** 10.1093/gbe/evu234

**Published:** 2014-10-27

**Authors:** Chao Xu, Jigang Zhang, Yu-Ping Wang, Hong-Wen Deng, Jian Li

**Affiliations:** ^1^Center for Bioinformatics and Genomics, Department of Biostatistics and Bioinformatics, School of Public Health and Tropical Medicine, Tulane University; ^2^Department of Biomedical Engineering, School of Science and Engineering, Tulane University; ^3^Third Affiliated Hospital, China Southern Medical University, Guang Zhou, 510000, P. R. China

**Keywords:** chromosomal translocation, next-generation sequencing, recurrent translocation, structural variation

## Abstract

As an important subtype of structural variations, chromosomal translocation is associated with various diseases, especially cancers, by disrupting gene structures and functions. Traditional methods for identifying translocations are time consuming and have limited resolutions. Recently, a few studies have employed next-generation sequencing (NGS) technology for characterizing chromosomal translocations on human genome, obtaining high-throughput results with high resolutions. However, these studies are mainly focused on mechanism-specific or site-specific translocation mapping. In this study, we conducted a comprehensive genome-wide analysis on the characterization of human chromosomal material exchange with regard to the chromosome translocations. Using NGS data of 1,481 subjects from the 1000 Genomes Project, we identified 15,349,092 translocated DNA fragment pairs, ranging from 65 to 1,886 bp and with an average size of approximately 102 bp. On average, each individual genome carried about 10,364 pairs, covering approximately 0.069% of the genome. We identified 16 translocation hot regions, among which two regions did not contain repetitive fragments. Results of our study overlapped with a majority of previous results, containing approximately 79% of approximately 2,340 translocations characterized in three available translocation databases. In addition, our study identified five novel potential recurrent chromosomal material exchange regions with greater than 20% detection rates. Our results will be helpful for an accurate characterization of translocations in human genomes, and contribute as a resource for future studies of the roles of translocations in human disease etiology and mechanisms.

## Introduction

Chromosomal translocation, a common type of structural variants (SVs), alters a genome through a whole chromosome or chromosomal segment attachment or interchange. Translocation may disrupt gene structures, and consequently gene functions. Recent studies have showed that translocations, especially those with even exchange of materials or balanced translocations are associated with various diseases, such as infertility and cancer (Aplan 2006; Sandberg and Meloni-Ehrig 2010; Mikelsaar et al. 2012). Thus it is important to characterize translocations and understand their roles in the disease etiology.

Various techniques and approaches have been developed for identifying chromosomal translocations. Examples include karyotyping by G-banding techniques based on morphological similarity (Drets and Shaw 1971); fluorescence in situ hybridization (FISH) (Bauman et al. 1980), such as M-FISH based on DNA sequence homology (Speicher et al. 1996); and array-based methods, such as array comparative genomic hybridization (array-CGH) (Solinas-Toldo et al. 1997). Although great progress has been made recently, the resolutions of these techniques are generally still in the magnitudes of hundreds or more base pairs (Le and Gribble 2012; Askree et al. 2013). In addition, these techniques can be labor intensive and be limited by the experiment materials such as the customized arrays (Le and Gribble 2012). Recently, next-generation sequencing (NGS) data are used for chromosome rearrangement analyses. This produced SV detection in a high-throughput and high resolution manner, allowing identifying and characterizing breakpoints and translocated fragments for translocations in a much refined scale than before (Hayes and Li 2013; Ordulu et al. 2014).

Only a few studies have been conducted for characterizing chromosomal translocations on the human genome using NGS data, and they are focused on mechanism-specific or site-specific translocation mapping. For example, Ou et al. (Ou et al. 2011) studied the nonallelic homologous recombination (NAHR)-mediated translocation and provided a computationally determined genome-wide “recurrent translocation map.” Other studies mapped oncogene-specific translocations in the mouse genome, revealing that oncogenic patterns of translocations might be an intrinsic feature of the translocation process (Burgess 2011; Chiarle et al. 2011; Klein et al. 2011). These studies provided insights into our understanding of certain-specific chromosomal translocations. However, in order to obtain a broad picture of the translocations on the human genome and along with the importance of translocations in human disease etiology, studies for providing a general genomic characterization of the chromosomal translocations are needed (Baker 2011).

In this study, using the NGS data from the 1000 Genomes Project (Abecasis et al. 2012), we had conducted a comprehensive analysis to provide a genome-wide characterization of chromosomal material exchange with regard to the chromosome translocations, and had identified and studied a number of translocation hot regions/pairs. This study will be helpful for our understanding of the general distributions and characteristics of the chromosomal translocations.

## Materials and Methods

### Data Sources

The low-coverage whole-genome sequence data (assembled based on the Human Reference Genome GRCh37/hg19) from the 1000 Genomes Project (http://www.1000genomes.org/home, last accessed October 29, 2014) were used in our study. The data included 1,481 subjects from five ethnic groups with a total of 26 subgroups: 340 East Asian subjects, 238 African subjects, 602 European subjects, 224 admixed American subjects, and 77 South Asian subjects. The detailed information for the data and study subjects can be found at http://www.1000genomes.org/about#ProjectSamples (last accessed October 29, 2014).

### Translocation Detection

SVDetect (v0.7) was used to detect the translocated fragments from the NGS data (Zeitouni et al. 2010). This software is designed to identify genomic structural variations from paired-end and mate-pair NGS data produced by various platforms. Applying both sliding window and clustering strategies, SVDetect uses anomalously mapped read pairs provided by short read aligners to localize genomic rearrangements and classify their types, for example, large insertions–deletions, duplications, and balanced or unbalanced chromosomal translocations. Default software parameters were used in our analytical process, except for customizing values of sliding window size for each subject in order to detect large and balanced translocations.

### Quality Control and Filtering

In order to control the false positives due to alignment errors, we employed two data filtering strategies, which have been proved to be very successful in removing false positive calls (Mijuskovic et al. 2012). The first strategy was a simple repeat filter for removing the translocations overlapping with repetitive regions, and the second strategy was the low mappability regions filter to remove the translocations falling into the low mappability regions (genomic regions tending to produce ambiguous mapping). Information for simple repeat regions and low mappability regions was obtained from UCSC Table Browser (Karolchik et al. 2004). The simple repeat regions were annotated as simple tandem repeats (possibly imperfect repeats) located by Tandem Repeats Finder (Benson 1999). There were several options of the low mappability filters based on the length of the mapping sliding windows of k-mers (where k had been set to 36, 40, 50, 75, or 100 nt) (Derrien et al. 2012). A smaller k-mers would result in larger low mappability regions. With read lengths of the sequencing data ranging from 65 to 130 bp and averaging at approximately 95 bp, a stringent filter—the low mappability region generated based on 75mer—was used. Intra and interchromosome translocations were removed when they overlapped with the low mappability regions at least 85% and 50%, respectively. The process was implemented using our own scripts.

### Comparison with Previously Available Translocation Data

Previously available translocation data were obtained from a number of public databases:

- *dbCRID* (Database of Chromosomal Rearrangements In Diseases, http://dbCRID.biolead.org, last accessed October 29, 2014): a comprehensive database of human chromosomal rearrangements and their associated diseases (Kong et al. 2011).
- *DACRO* (Disease-Associated Chromosomal Rearrangements Online, https://www1.hgu.mrc.ac.uk/Softdata/Translocation/, last accessed October 29, 2014): a simple, searchable database of all published chromosomal rearrangements that are associated with an abnormal phenotype. Its records can be ascertained through online searches of PubMed, SCOPUS, and OMIM.
- *TICdb* (http://www.unav.es/genetica/TICdb/, last accessed October 29, 2014): a database of translocation breakpoints in cancer, containing greater than 1,300 fusion sequences found in human tumors and involving greater than 400 genes (Novo et al. 2007).

Translocation data extracted from these databases were manually curated by removing missing and ambiguous data. The curated data, along with low-copy repeat (LCR) substrate data from another study (Ou et al. 2011), were then used for comparison with our results. Note that the data from dbCRID and DACRO were presented on chromosome-band levels, whereas LCR substrate data and our study were presented on nucleotide level. To facilitate comparison, data presented on chromosome-band levels were transformed into the base pair scales using UCSC cytoband annotation files (Karolchik et al. 2014).

### Translocation Hot Region Characterization and Annotation

Translocation hot regions referred to chromosomal regions with high translocation occurrence. In our study, we defined a region as translocation hot region when the translocation occurrence in the region was greater than 1,000 (supplementary fig. S1, Supplementary Material online). Circos (Krzywinski et al. 2009) was employed to plot the translocations in these hot regions (supplementary figs. S2 and S3, Supplementary Material online).

Annotations for genes and coding regions in the translocation hot regions were based on annotation files obtained from NCBI (seq_gene.md vDec12 and CCDS.20130430.txt). Annotations for the genomic variations (Iafrate et al. 2004) and repetitive elements were conducted through the UCSC Genome Browser. G-banding annotation was from the UCSC table “cytoband.txt” with eight types of bands: gpos100, gpos75, gpos50, gpos25, gneg, acen, gvar, and stalk (Karolchik et al. 2004). Note that gpos100, gpos75, gpos50, and gpos25 are classes containing progressively lighter staining G-positive bands, and gneg class consists of the nonstaining G-negative light bands (Furey and Haussler 2003). Python, Bash, and MySQL scripts were prepared to facilitate the data analysis.

### Enrichment Scores

To measure the number of translocations between two specific chromosomes, enrichment scores, normalized by their sizes and proposed in Lieberman-Aiden et al. (2009) and Duan et al. (2010), were used. They were computed as ratios between the observed and expected numbers of translocations for a pair of chromosomes:

$$\frac{{\text{N}}_{i,j}}{\frac{{\text{N}}_{i}}{\text{N}}\times \frac{{\text{N}}_{j}}{\text{N}}\times \text{N}},$$

where N*_i_*_,_*_j_* is the number of observed translocated fragment pairs between chromosomes *i* and *j*, N*_i_* (N*_j_*) is the number of observed interchromosome pairs involving chromosome *i*(*j*), and N is the total number of all the observed interchromosome pairs.

### Motif Search

Motif search for common molecular features was conducted through MotifSearch.Com, an online motif search tool built on the state-of-the-art qPMS7 algorithm (Dinh et al. 2012). It adapted one of the most well-known motif search model—Planted Motif Search (Rajasekaran 2009) which can precisely capture the nature of motifs and usually give the most accurate results.

Both the translocation hot regions and motifs were investigated for their evolutionary conservation. For translocation hot regions, we used phastCons and phyloP scores in UCSC Genome Browser (Siepel et al. 2005; Pollard et al. 2010). For the motifs, their sequences were searched in cisRED, a database holding “conserved sequence motifs identified by genome-scale motif discovery, similarity, clustering, cooccurrence, and coexpression calculations” (Robertson et al. 2006).

## Results

### Basic Characteristics of the Detected Translocations

Using SVDetect and analyzing the alignment files of the 1,481 NGS samples from the 1000 Genomes Project, 18,867,464 translocated fragment pairs were called. After filtering out the translocations overlapped with repetitive regions and low mappability regions (genomic regions tending to produce ambiguous mapping), 15,349,092 pairs, including 16,432 (0.11%) intrachromosome pairs, were remained and used for all the subsequent analyses.

**Fig. 1.—:**
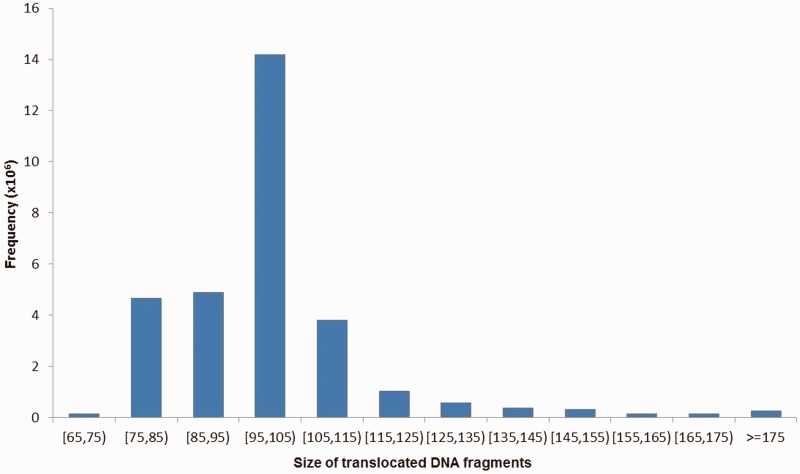
Size distribution of the identified translocated DNA fragments. *X* axis indicates the size groups of the identified translocated DNA fragments. *Y* axis represents the corresponding frequencies.

**Fig. 2.—:**
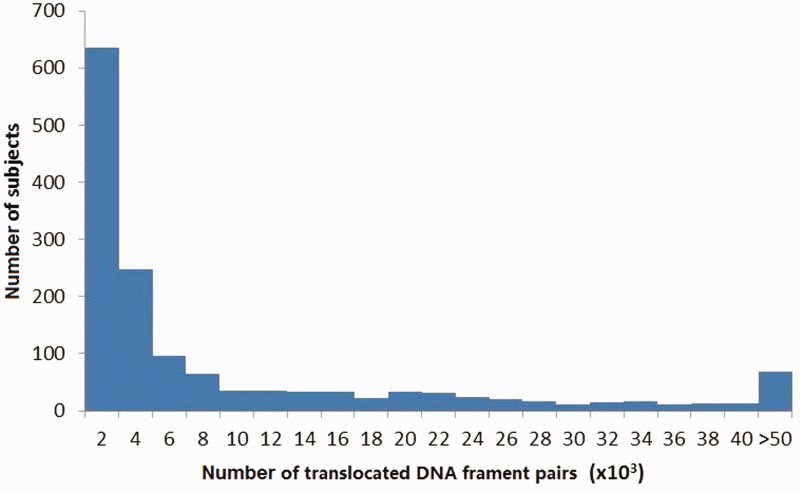
The numbers of translocated DNA fragment pairs on individual study subjects. *X* axis indicates the number of translocated DNA fragment pairs per subject. *Y* axis indicates the number of subjects.

The size distribution of all translocated fragment pairs was shown in figure 1, ranging from 65 to 1,886 bp with an average of approximately 102 bp and a median of 101 bp. In total, 4,895 pairs were greater than 1 kb, which we defined as >1 kb translocations. The numbers of translocated fragment pairs on individual study subjects were shown in figure 2, with 95.0% of these numbers ranging from 177 to 49,235 and an average of 10,364 pairs per subject. The detected translocated fragments covered about 57.3% of the whole genome (including both autosomal and X chromosomes). For regions covered by the translocated fragments, approximately 44.9% were genic regions and approximately 34.5% were coding sequence regions (based on NCBI annotations, December 2012 version), consistent with results from previous studies (Chiarle et al. 2011). Approximately 52.9% of the translocated fragments overlapped with G-positive regions. For each subject, approximately 0.069% of his/her genome were covered by translocated fragments.

### Recurrent Translocations

Several recurrent translocations have been previously described in humans (Ou et al. 2011), such as t(11;22)(q23;q11), t(8;22)(q24.13;q11.21), and t(4;8)(p16;p23). Usually, the nomenclature of translocations uses the cytoband as the basic unit. For the large amount of small size translocated fragments in our result, the cytoband unit is too large to make a compatible comparison. However, small size translocations in all the three regions were observed in our results, which showed chromosomal material exchange in those regions. Greater than 1 kb translocations were also found in the region of t(4;8)(p16;p23). Table 1 summarized the detection rate of the translocated fragment pairs in the regions of three well known and three recently proposed recurrent translocations. The detection rate in five of the six above mentioned regions showed ethnic differences in our results (*P* values ranging from 3.64 × 10^−^^12^ to 4.29 × 10^−^^2^). These differences may be due to different demographic histories of the study samples.

**Table 1:** Detection Rates of Chromosomal Material Exchanges in Known Recurrent Constitutional Translocations in Different Ethnic Groups

Ethnic Group	t(11;22) (q23;q11)	t(8;22) (q24.13;q11.21)	t(4;8) (p16;p23)	t(4;11) (p16.2;p15.4)	t(4;8) (p16.2;p23.1)	t(8;12) p23.1;p13.31)
AFR (%)	18.07	6.72	53.36	1.68	2.94	15.55
AMR (%)	20.54	4.46	45.98	2.68	1.79	14.29
ASN (%)	15.88	2.65	36.76	2.35	2.35	11.47
EUR (%)	16.61	4.15	36.71	3.32	1.50	12.46
SAN (%)	18.18	5.19	35.06	0.00	6.49	15.58
All (%)	17.35	4.32	40.72	2.57	2.23	13.17
*P* value	0.1279	7.3 × 10^−4^	3.64 × 10^−12^	1.86 × 10^−6^	4.55 × 10^−11^	0.0429

Note.—AFR, Africa; AMR, admixed American; ASN, East Asian; EUR, European; SAN, South Asian; “All” is the sample with AFR, AMR, ASN, EUR and SAN combined; “*P* value” is the chi-square test *P* value for the rate differences among different populations.

To study recurrent translocated fragments, the whole genome was divided into small bins (200 bp each or about 2-fold of the average transloction size) and translocations between different bins were considered. Through this, additional regions with recurrent chromosomal material exchange not mentioned in previous studies were identified. The top five bin pairs were shown in table 2, each observed in greater than 20% of study subjects. Note that all the bins for region 1 and 5 were located in G-banded regions. In addition, regions with recurrent chromosomal material exchange observed in more than 15 subjects (detection rate > 1%) overlapped with approximately 5.14% (99/1,925) translocations characterized in available disease-related translocation databases, although they may not be indicated as recurrent translocations in these databases.

**Table 2:** Top 5 Novel Regions of Recurrent Chromosomal Material Exchange

Region	Chr	Begin	End	gieStain	Gene	Chr	Begin	End	gieStain	Gene	Detection Rate
1	12	82958601	82958800	gpos100		16	14580801	14581000	gpos50	*PARN*	28.6
2	6	382201	382400	gneg		16	33428201	33428600	gneg		26.4
3	3	49783601	49783800	gneg	*IP6K1*	5	87153001	87153200	gpos100		25.7
4	21	11022401	11022600	acen	*BAGE2*	21	45991601	45991800	gneg	*TSPEAR*	23.0
5	11	61841601	61841800	gpos25		14	81786801	81787000	gpos100	*STON2*	21.1

Note.—Chr, chromosome; gieStain, Giemsa stain results: acen, pericentromeric region; gpos100 class consists of the darkest staining bands, with gpos75, gpos50 and gpos25 classes containing progressively lighter staining G-positive bands; gneg class consists of the nonstaining G-negative light bands (Furey and Haussler 2003).

### Translocation Hot Region Characterization and Annotation

Translocated fragments were found to occur in high rates in a number of genomic regions, with the top 16 regions with the occurrence greater than 1,000 shown in table 3. The full set of translocated fragments for these 16 regions is included in the supplementary data set S1, Supplementary Material online. The distribution of the occurrence along the whole genome is shown in supplementary figure S4, Supplementary Material online. Ten of 16 hot regions were located in G-negative regions, while the remaining six were in G-positive regions. Fifteen of these 16 regions were previously reported in the Database of Genomic Variants (Macdonald et al. 2014) for containing structural variations such as inversion, indel and copy number variation. Fourteen of these 16 regions contained repetitive elements, such as short interspersed nuclear elements, long interspersed element (LINE)-mediated retrotransposition, or simple repeats. Note that LINE-mediated retrotransposition has been proposed as a major mechanism for human genome translocations (Liu et al. 2012). The two regions not containing repetitive elements were chr9:140785301–140785680 (fragment A) and chr6:382041–382470 (fragment B). Based on the annotation from UCSC Genome Browser (Karolchik et al. 2014), both of them showed DNase I hypersensitivity and might be targeted binding sites for *NRSF*, *NFKB*, and *c-Myc.* Interestingly, *c-Myc* was mentioned to participate in recurrent oncogenic translocations in B cell lymphomas (Chiarle et al. 2011).

**Table 3:** Annotation of the Top 16 Translocation Hot Regions

Hot Region	Chromosome	Begin	End	Size	gieStain	CDS	Gene	Repeats	Occurrence
1	12	66451361	66451530	170	gpos50			SINE, Simple	8627
2	7	105741881	105741990	110	gneg	CCDS47685.1	*SYPL1*	Simple	3911
3	2	33141301	33141770	470	gpos75		*LINC00486*	Simple	2833
4	6	160521751	160521890	140	gneg	CCDS5273.1	*IGF2R*	SINE, LINE	2700
5	1	231007791	231007920	130	gpos50			SINE, LTR	2683
6	2	88206871	88207030	160	gneg			LINE, Simple	1936
7	2	238252121	238252260	140	gneg	CCDS33412.1	*COL6A3*	SINE, Simple	1642
8	9	140785301	140785680	380	gneg	CCDS59523.1	*CACNA1B*		1513
9	6	382041	382470	430	gneg				1371
10	3	64682161	64682300	140	gpos50		*ADAMTS9-AS2*	SINE	1262
11	4	1708921	1709060	140	gneg	CCDS3350.1	*SLBP*	SINE	1206
12	1	62390831	62390970	140	gpos50	CCDS617.2	*INADL*	SINE, Simple	1193
13	17	30276681	30276790	110	gneg	CCDS11270.1	*SUZ12*	SINE	1127
14	16	33428141	33428570	430	gneg			LINE	1116
15	5	141379101	141380040	940	gneg			SINE, LINE	1083
16	7	107410631	107410760	130	gpos75	CCDS5748.1	*SLC26A3*	SINE, Simple	1067

Note.—CDS, coding sequence id in NCBI; Repeats, repetitive elememts contained in the region including simple repeats (Simple); SINE, short interspersed nuclear elements; LINE, long interspersed nuclear elements; LTR, long terminal repeat elements; occurrence, the numer of observations of translocations in the region.

The fragments originated from/translocated into these translocation hot regions were dispersed differently on the genome. For most of the regions containing repetitive elements, the translocated fragments were approximately randomly spread across the genome. For the two regions without repetitive elements (fragments A and B) and region chr16:33428141–33428570 (fragment C), the translocated fragments were concentrated in certain regions of the genome (supplementary figs. S2 and S3, Supplementary Material online). Especially, the translocations in fragment B and fragment C were mainly between these two regions. In addition, translocations between fragment B and fragment C were observed in 1,139 (76.9%) of the 1,481 study subjects, illustrating the existence of a recurrent translocation.

The three fragments were found to have a low probability to be evolutionary conserved. For example, the phastCons scores for the three fragments are 0.024, 0.002, and 0.014, respectively. Note that, phastCons scores estimate the probability that each nucleotide belongs to a conserved element, and thus are between 0 and 1 with greater values indicating greater conservation. Similar conclusion can be drawn using phyloP scores (supplementary table S1, Supplementary Material online). This observation is consistent with the fact that these regions are “hot” with increased chances of DNA material exchanges.

To further explore the common molecular features of translocation hotspots, we analyzed the occurrences of hotspot motifs in those regions. A 10-mer common sequence motif (5′-CCCAGGCTGG-3′) was found in approximately 75% of the hot regions, including the two regions not containing repetitive elements. This motif has been indicated to be a potential transcriptional element (Volinia et al. 1992). When searched in cisRED, this 10-mer sequence was found being fully contained in seven 11-bp motifs. All of these seven motifs were in the promoter regions with discovery *P* values less than 0.05 (supplementary table S2, Supplementary Material online), further indicating the potential functional importance of the identified motif.

### Functional Analysis

Gene ontology (GO) analyses were conducted using WEB-based GEne SeT AnaLysis Toolkit (WebGestalt) (Zhang et al. 2005; Wang et al. 2013) for the genes with top 5% translocation observations. GO terms containing at least 15 and no more than 250 genes were considered. The significantly enriched GO terms were presented in table 4. Molecular function of structural constituent of ribosome, and biological process related to translation initiation, cellular macromolecular complex disassembly were most enriched.

**Table 4:** GO Analysis for the Genes with Top 5% Translocation Occurrences

Category	GO Term	GO ID	C	O	E	R	rawP	adjP
Biological Process	Translational initiation	GO:0006413	152	12	2.79	4.29	2.42 × 10^−5^	0.0142
	Cellular macromolecular complex disassembly	GO:0034623	177	13	3.25	3.99	2.41 × 10^−5^	0.0142
	Macromolecular complex disassembly	GO:0032984	182	13	3.35	3.88	3.23 × 10^−5^	0.0142
	Nuclear-transcribed mRNA catabolic process, nonsense-mediated decay	GO:0000184	119	10	2.19	4.57	6.84 × 10^−5^	0.0226
	Cellular protein complex disassembly	GO:0043624	156	11	2.87	3.83	0.0001	0.0264
	Serine family amino acid metabolic process	GO:0009069	31	5	0.57	8.77	0.0002	0.0378
	Protein complex disassembly	GO:0043241	161	11	2.96	3.72	0.0002	0.0378
Molecular Function	Structural constituent of ribosome	GO:0003735	157	14	3.04	4.6	2.24 × 10^−6^	0.0006
	Methyltransferase activity	GO:0008168	188	12	3.64	3.3	0.0003	0.0207
	Immunoglobulin binding	GO:0019865	19	4	0.37	10.87	0.0004	0.0207
	Transferase activity, transferring one-carbon groups	GO:0016741	194	12	3.76	3.19	0.0004	0.0207
	mRNA binding	GO:0003729	91	7	1.76	3.97	0.0019	0.0492

Note.—C, the number of reference genes in the GO term; O, the number of genes in the gene set and also in the GO term; E, expected number in the GO term; R, ratio of enrichment (O/E); rawP, *P* value from hypergeometric test; adjP, *P* value adjusted by the multiple test adjustment.

### Interchromosome Translocation Occurrence

**Fig. 3.—:**
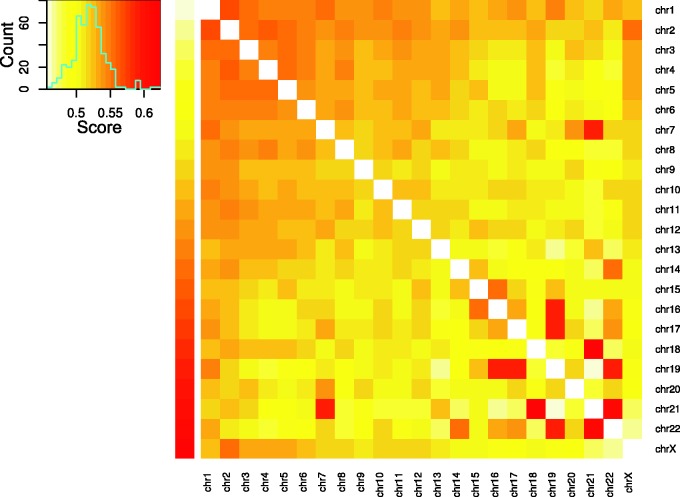
Heat map of the interchromosomal translocated fragment pairs. The color key changes from light yellow (low enrichment scores) to red (high scores). The upper left panel is the histogram of the enrichment scores. The cell in the heat map shows the interchromosomal translocated fragments enrichment score between the corresponding chromosomes.

The chances that translocations were between two specific chromosomes were not the same for different chromosome pairs. Based on the enrichment score defined in the Materials and Methods section, the enrichment degree of interchromosome translocated fragments among chromosome pairs was shown in figure 3. Similarly to the previously detected patterns (Lieberman-Aiden et al. 2009), relatively more translocations were observed between small and gene-rich chromosomes 16, 17, 19–22. Furthermore, previous FISH studies on chromosome territories showed that chromosomes 16, 17, 19, 22 were concentrated together in the center of the nucleus (Boyle et al. 2001; Cremer and Cremer 2010). The enrichment scores of these chromosome pairs clearly reflected their spatial proximity of chromosome territories in cell nuclei. Particularly, the top three chromosomal pairs with most enriched translocations were t(18,21), t(21,22), and t(17,19). Notice that chromosome 18 is not gene-rich and is not close to those gene-rich chromosomes in space (Tanabe et al. 2002; Cremer and Cremer 2010). However, other studies (Lieberman-Aiden et al. 2009) did provide evidence that chromosomes 18 and 21 may contact each other with high probabilities.

### Effects of Gender

Among the 1,481 study subjects, 1,147 had gender information. When tested, no significant difference was found for the translocation occurrence rates in males and in females (*t*-test, *P* value = 0.276). The effects of gender on translocation rates among healthy controls obtained here is similar to those detected by cytogenetic methods (Whitehouse et al. 2005; Sigurdson et al. 2008), although the resolution would be very much different.

## Discussion

Our study depicted the characteristics of the human chromosomal material exchange with regard to the chromosome translocations using NGS data from the 1000 Genomes Project. Compared with traditional methods, translocation detection techniques using NGS data will provide results in high resolutions. In our results, the average length of translocated fragments was about 102 bp, a much more refined resolution than that of the traditional methods. The increased resolutions will allow the detection of small size translocations on the human genome, which have not been well characterized previously. For example, our study identified an average number of 10,364 translocated fragment pairs per subject, 99.3% of which are less than 200 bp, indicating new perspectives on human translocations provided by NGS data.

In our study, 4,895 translocated fragment pairs with size being greater than 1 kb were identified. This is about 0.03% of all the detected translocations, indicating NGS data may have limited power to detect large size translocations. This may be partially due to two reasons. First, the insert size of the paired-end sequencing data limits the sizes of identified SVs (Medvedev et al. 2009). In the sequencing data we used, the average insert size is about 300 bp. Second, the sliding window sizes in SVDetect will limit the sizes of the identified translocated fragments. SVDetect manual recommends a window size at least $2\mu +2\sqrt{2\sigma }$ to identify large and balanced translocations, where the μ and σ are the mean and standard deviation of the insert size, respectively (Zeitouni et al. 2010). In our study, we used the above-mentioned recommended value for the sliding window size, which is probably conservative. Thus, multiple libraries with varying insert sizes and multiple SVDetect parameter settings may be necessary to discover the whole size range of translocations and other SVs (Medvedev et al. 2009).

The quality of the detected translocations using NGS data may be affected by the NGS alignment errors and duplicate callings. Systematic alignment errors may be introduced by reads originating from repetitive regions. To reduce such effects and control false positive detections, we have followed suggestions on data filtering and parameter settings provided in a recently developed analysis pipeline for the detection of SVs including translocations using NGS data (Mijuskovic et al. 2012). Through the validation by polymerase chain reaction using customized primers, they showed that filtering SV calls against low mappability regions can successfully remove false positive calls. Their filtering parameters, such as the overlapping rate of 85% and 50% for intra and interchromosome translocations, were determined to achieve an acceptable rate of false positives versus false negatives. Applying this filter together with other controlling strategies including filtering out simple repeats and duplicate records, approximately 18.7% translocation calls in our result were filtered out, likely resulting in a more reliable translocation set.

Different types of study subjects in terms of disease status have been used for translocation studies. For example, the three databases used in our analyses, dbCRID (Kong et al. 2011), DACRO (see Materials and Methods), and TICdb (Novo et al. 2007), are all associated with human diseases such as cancers. On the other hand, the study subjects in our sample are generally healthy. When results are compared, approximately 76–90% of the characterized translocations in the disease-associated databases overlapped with our results, indicating the power and usefulness of translocation detection through NGS data. However, the detection rates of these overlapping translocations are quite low: greater than 95% with less than 7% detection rate with an average of approximately 2.7%, probably an indication of diverse occurrence rates for certain translocations for healthy and nonhealthy individuals. When both case and control samples are available, the characterization of translocation could be improved with better accuracy (Mijuskovic et al. 2012). Thus, our results could potentially provide complementary information to previously available data and serve as a control set resource for the detection of the disease-related translocations.

Several mechanisms for human genome translocations have been summarized by Liu et al. (Liu et al. 2012), including LINE-mediated retrotransposition, LCR mediated NAHR and so on. The identified translocated fragments may be related to various translocation mechanisms. For example, in our result, approximately 16.8% of the identified translocations and approximately 17.8% of the greater than 1 kb translocations at least 50% overlapped with LINE sequences. Four of the 16 translocation hot regions were found to start or end within LINE sequences. These translocations are probably caused by the LINE-mediated mechanism. For the LCR mediated NAHR translocations, a previous study has provided a genome-wide map of LCR substrates (Ou et al. 2011). Approximately 86.9% of those reported substrates were fully covered by our results, corresponding to approximately 1.10% of our identified translocations and approximately 13.5% of the greater than 1 kb translocations. For the remaining identified translocated fragments, however, whether they are related to other known or unknown translocation mechnaisms remains to be evaluated.

The distribution of translocated fragments along the genome is not uniform. When categorized based on the Giemsa stain annotation, the fragments were enriched in G-positive regions (Fisher’s exact test *P* value 5.26 × 10^−^^8^). Specifically, the fragments were enriched in the darker G-staining regions gpos100 and gpos75, with *P* values of 9.91 × 10^−^^10^ and 5.80 × 10^−^^4^, respectively. However, when only translocation hot regions were considered, they were not enriched in G-positive regions. In addition, previous studies (Chiarle et al. 2011) found translocations of certain mechanisms were enriched in different types of G-bands. Thus, the genome-wide distribution trend for translocated fragments will be a mixture of translocations due to all the known and unknown mechanisms. Given the unique features of different types of G-bands, such as different GC contents, structures, and transcription activities, it may be an indication that chromosome features may have various effects on translocations of different mechanisms.

## Conclusions

In summary, we have conducted a comprehensive analysis of human chromosomal material exchange with regard to the chromosome translocations, using the NGS data from the 1000 Genomes Project. Through this study, we have characterized genome-wide translocated DNA fragments/pairs in terms of sizes and numbers, and identified novel recurrent chromosomal material exchange regions and translocation hot regions/pairs. Our results will contribute as a resource for future chromosomal translocation studies, particularly, for studying their roles in human disease etiology and mechanisms.

## Supplementary Material

Supplementary data set S1, figures S1–S4, and tables S1 and S2 are available at *Genome Biology and Evolution* online (http://www.gbe.oxfordjournals.org/).

Supplementary Data
