# Supplementary material for: Characterization of Human Chromosomal Material Exchange with Regard to the Chromosome Translocations Using Next-Generation Sequencing Data
Source: Genome Biol Evol. 2014 Oct 27;6(11):3015–24. doi: 10.1093/gbe/evu234 (PMC4255766; doi:10.1093/gbe/evu234)
Supplement: Supplementary Data [file supp_6_11_3015__index.html]

Supplementary Data 

# Characterization of Human Chromosomal Material Exchange with Regard to the Chromosome Translocations Using Next-Generation Sequencing Data

## Supplementary Data

files

**Files in this Data Supplement:**

- Supplementary Data - xlsx file
- Supplementary Data - doc file
- Supplementary Data - xlsx file
