# Supplementary material for: Characterization of Human Chromosomal Material Exchange with Regard to the Chromosome Translocations Using Next-Generation Sequencing Data
Source: Genome Biol Evol. 2014 Oct 27;6(11):3015–24. doi: 10.1093/gbe/evu234 (PMC4255766; doi:10.1093/gbe/evu234)
Supplement: Supplementary Data [file supp_evu234_evu-234_Supplementary_Figures.doc]

**Supplementary Figures**

**Supplementary Fig S1**

The translocation frequency (depth) in the vicinity of the 16 hot regions: a) 16 plots using same y-axis scale; b) 16 plots using different y-axis scale.

**Supplementary Fig S2**

9 of 16 translocation hot regions and their corresponding fragments, listed from top to down and from left to right. Different color indicates regions in different chromosomes.

**Supplementary Fig S3**

The rest 7 of 16 translocation hot regions and their corresponding fragments, listed from top to down and from left to right. Different color indicates regions in different chromosomes.

**Supplementary Fig S4**

The translocation density along the whole genome (autosomal and X chromosome). Each chromosome was split into 100 intervals. The average translocation occurrence in each interval was plotted as the “density”. The two red lines in each sub-graph indicate the first and last 5Mb of each chromosome. The region between the two blue lines is the centromere of each chromosome.


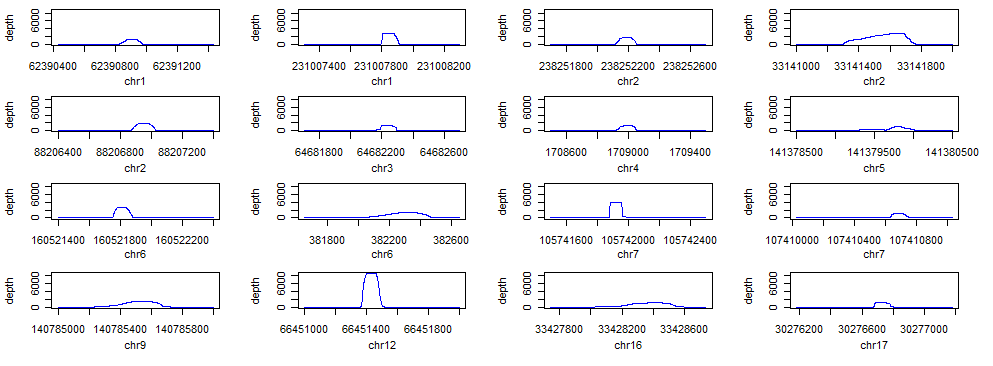


**a**


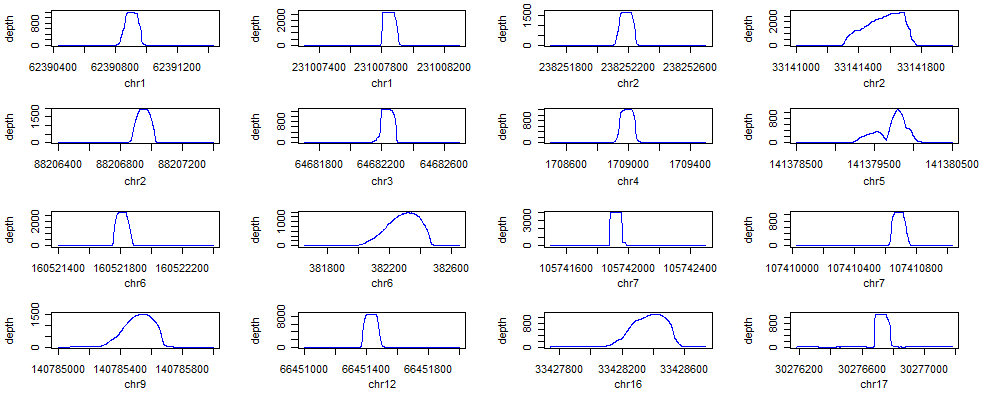


**b**

**Supplementary Fig S1**


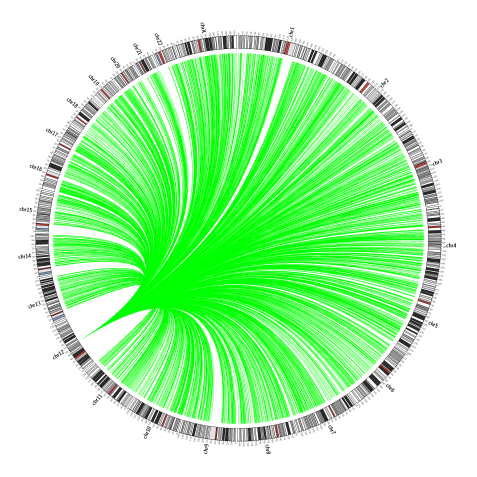

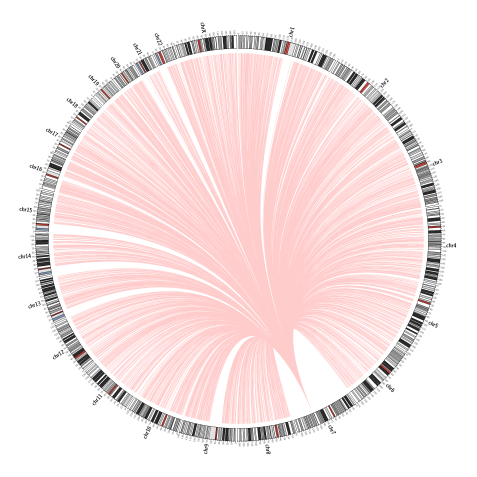

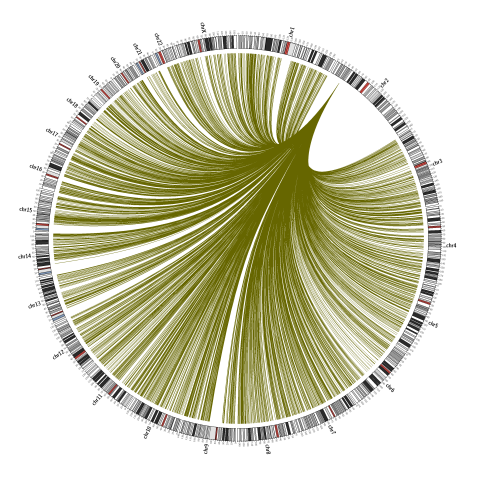

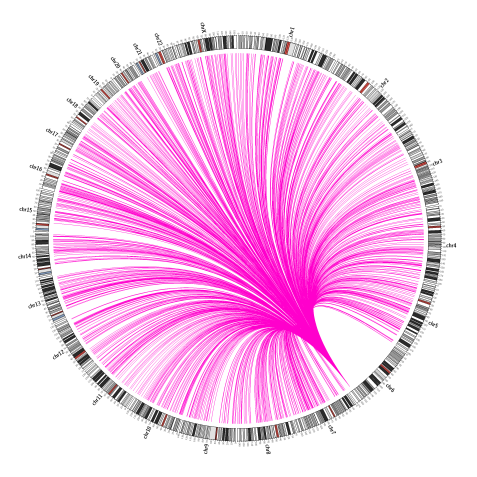

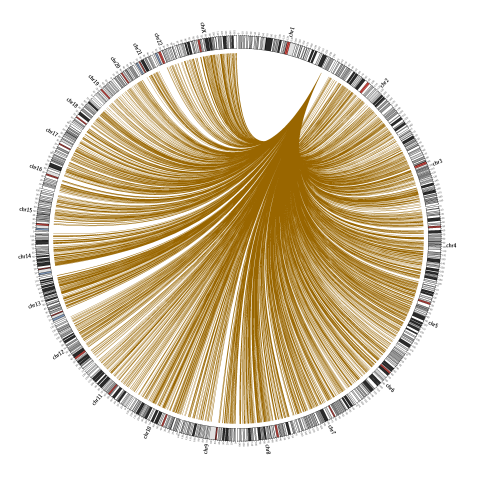

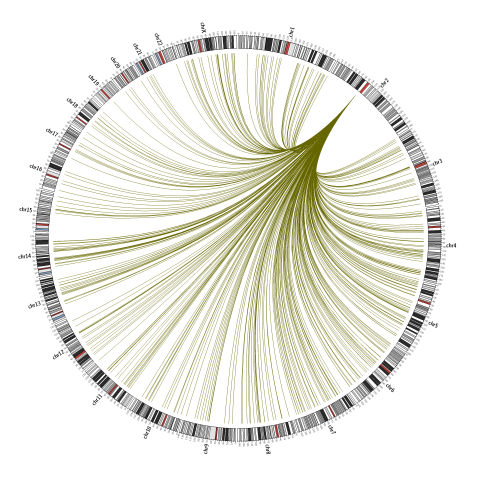


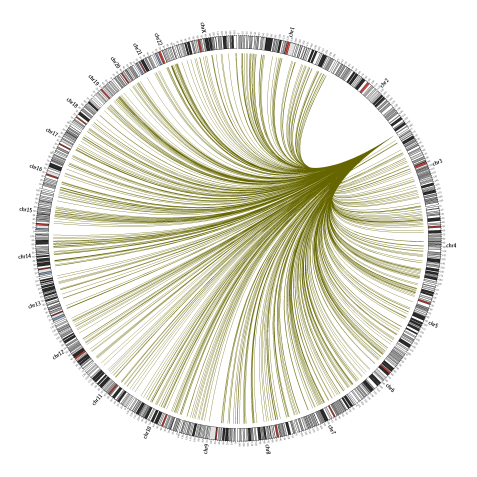

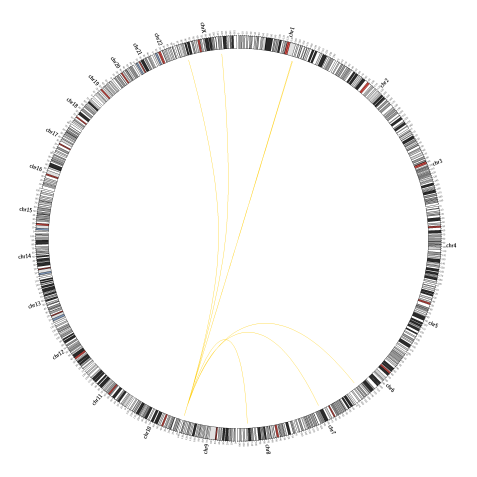

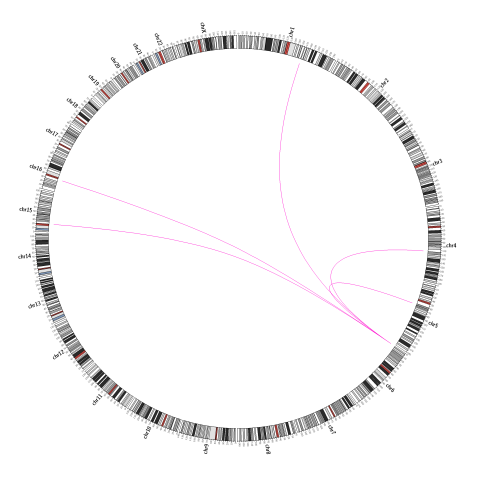


**Supplementary Fig S2**


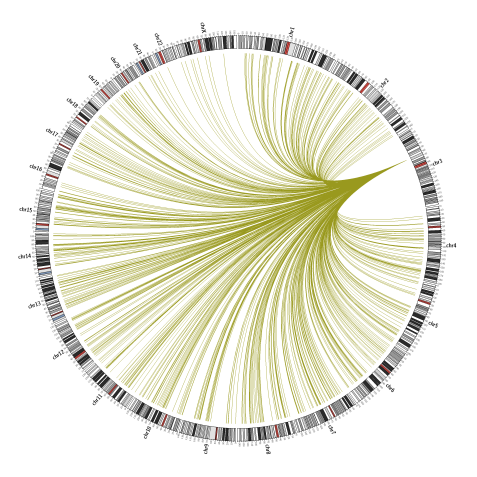

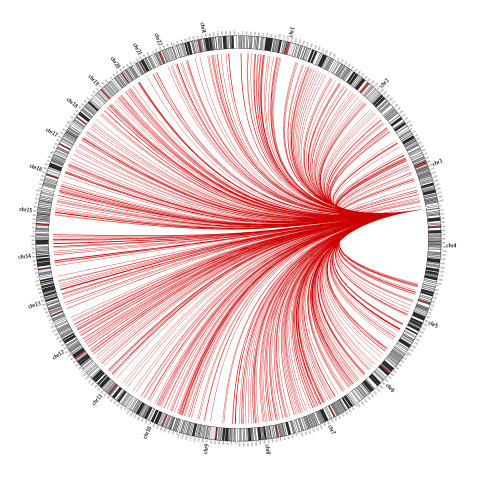

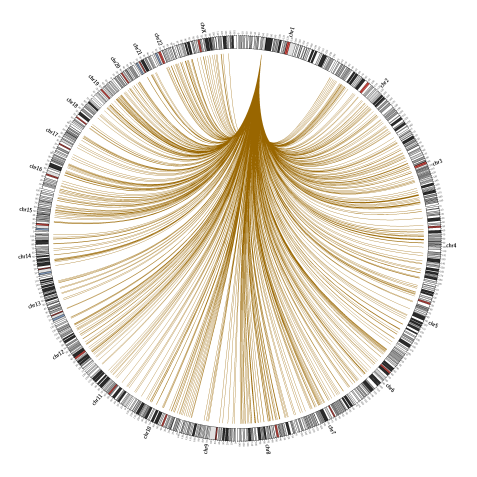

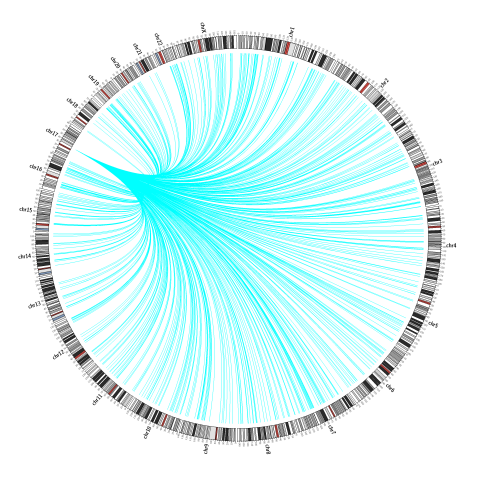

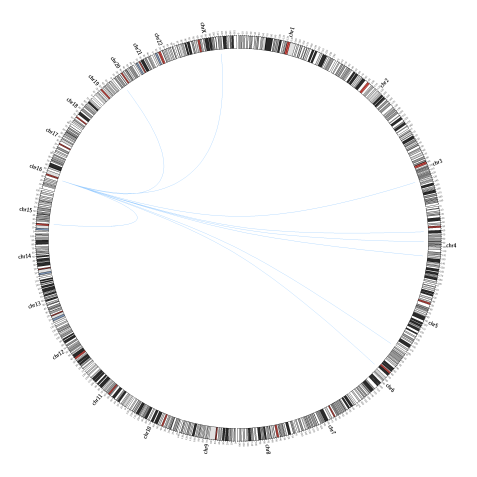

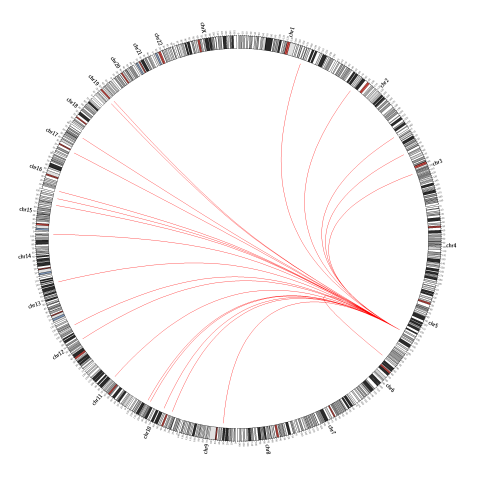

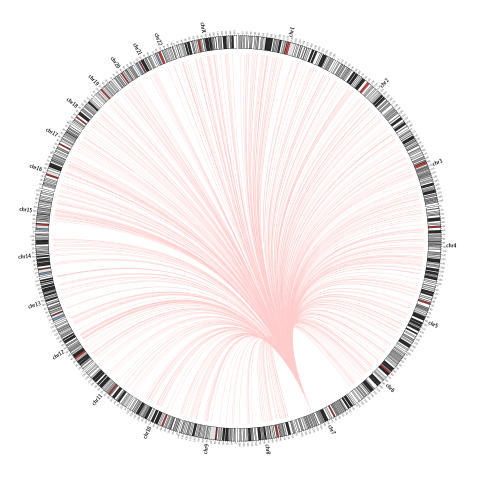


**Supplementary Fig S3**


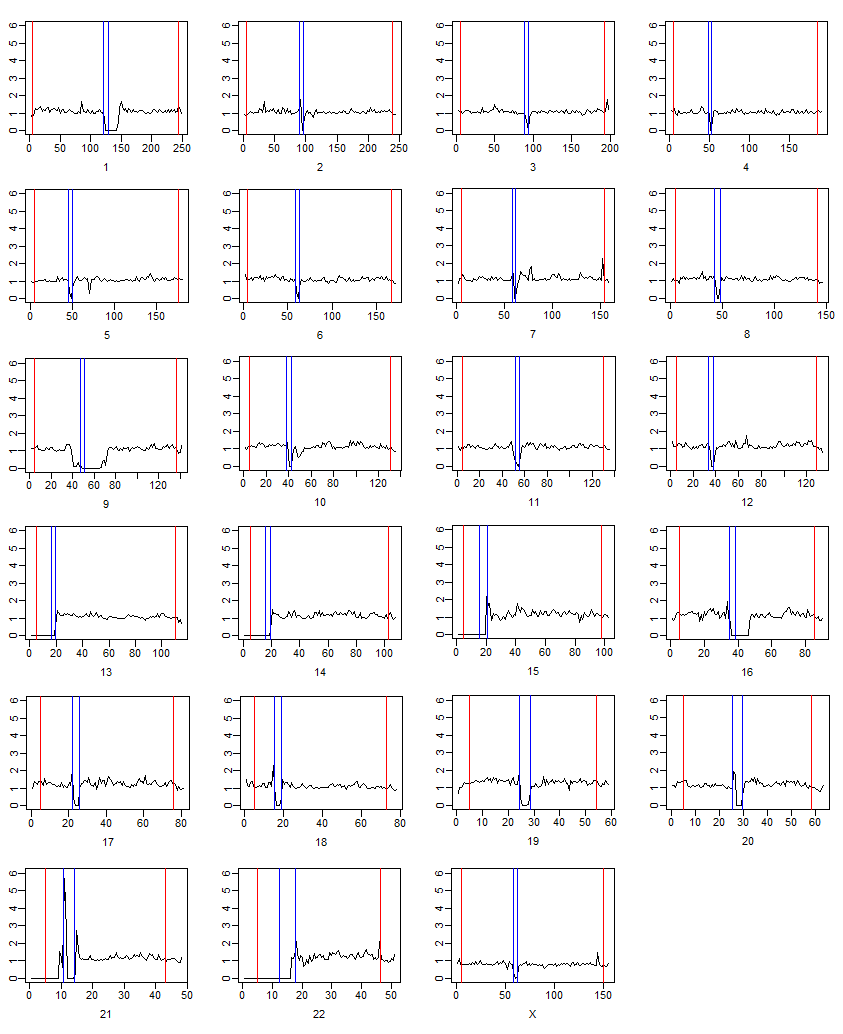


**Supplementary Fig S4**
